# Hiding in plain sight? A review of post-convulsive leukocyte elevations

**DOI:** 10.3389/fneur.2022.1021042

**Published:** 2022-11-02

**Authors:** Jose L. Vega, Barry R. Komisaruk, Mark Stewart

**Affiliations:** ^1^Department of Psychology, Rutgers University-Newark, Newark, NJ, United States; ^2^TeleNeurologia SAS, Medellin, Colombia; ^3^Department of Neurology, State University of New York Health Sciences University, Brooklyn, NY, United States; ^4^Department of Physiology and Pharmacology, State University of New York Health Sciences University, Brooklyn, NY, United States

**Keywords:** leukocytosis, leukocyte margination, seizure, SUDEP, neurogenic pulmonary edema, catecholamine, oxygen conserving reflex, leukocyte demargination

## Abstract

During physiological stress responses such as vigorous exercise, emotional states of fear and rage, and asphyxia, the nervous system induces a massive release of systemic catecholamines that prepares the body for survival by increasing cardiac output and redirecting blood flow from non-essential organs into the cardiopulmonary circulation. A curious byproduct of this vital response is a sudden, transient, and redistributive leukocytosis provoked mostly by the resultant shear forces exerted by rapid blood flow on marginated leukocytes. Generalized convulsive seizures, too, result in catecholamine surges accompanied by similar leukocytoses, the magnitude of which appears to be rooted in semiological factors such as convulsive duration and intensity. This manuscript reviews the history, kinetics, physiology, and clinical significance of post-convulsive leukocyte elevations and discusses their clinical utility, including a proposed role in the scientific investigation of sudden unexpected death in epilepsy (SUDEP).

## Introduction

For longer than a century, physicians have been routinely confronted with leukocytoses found unexpectedly in the bloodwork of patients who suffer one or more generalized convulsive seizures (GCSs). Aside from instigating infectious workups, these leukocytoses typically lack significance in patient care, probably because their physiological origins are incompletely understood. Some investigators have noted that the magnitude of post-convulsive leukocyte elevations (PoCLEs) bears a relationship with convulsive duration and severity. Others have noted the opposite. The paragraphs below present the history and pathophysiological context of PoCLEs and bring to light their potential role in the identification of patients at risk of sudden unexpected death in epilepsy (SUDEP), a complication of some epileptic seizures, typically GCSs, in which victims experience an acute and fatal respiratory collapse (1).

## Historical context

In 1896, Joseph Capps, then a house officer at the McLean Hospital in Boston, serendipitously noticed a marked leukocytosis in a patient who had suffered an epileptic convulsion just before his scheduled blood collection. Failing to find an infection, Capps correctly hypothesized that the leukocytosis had “depended” on the convulsion. To test this, he collected blood from “severely” epileptic patients as frequently as every four hours hoping that at least some baseline samples could be randomly collected just before the onset of a convulsion. When these samples materialized, he compared their leukocyte counts against new samples drawn immediately after the convulsions, and concluded that the observed leukocytoses (1) were induced by the convulsions, (2) were “… as sudden as [they were] pronounced…,” and (3) that their degree and duration correlated with the “length and severity of attacks” (2). Similar observations and conclusions were reported by others shortly after Capps' work was published (3–5).

The physiological explanation for PoCLEs as we understand them today was already underway during the fall of 1893 when the English physician George Oliver (1841–1915) fed his own son a glycerin extract made from sheep and calf adrenal glands, which constricted his radial arteries and accelerated his pulse. Oliver then enlisted Dr. Edward Shafer (1850–1935), a prominent physiologist at University College in London, to study the effects of his extract in animals (6), which led to a landmark paper that described, for the first time, the production of a “material” by the adrenal glands capable of maintaining and increasing vascular tone (7). By the turn of the twentieth century the Austrian scientist Otto Von Furth (1867–1938) obtained the extract's bioactive compound, “suprarenin,” and within 2 years, Jokichi Takamine (1854–1922) purified its crystalline form and named it adrenaline (8). Later, Walter Bradford Canon [1871–1945] discovered that emotions such as rage and fear led to a release of adrenaline, which accelerated the heart rate and redistributed blood flow from most organs toward the skeletal muscles, the heart, the lungs, and the brain: the fight or flight response (9). Cannon (9) and other investigators (10, 11) also reported an augmented release of adrenaline by asphyxia, which was said to dilate bronchial smooth muscles in order to allow for “a second wind” (9). Then, exogenous adrenaline (12), emotions of fear and rage (13), and physical exercise (14) were found to increase the peripheral leukocyte count [reviewed by Benschop et al. (15)]. Table 1 shows an approximate timeline of findings related to PoCLEs.

**Table 1:** Representative publications related to post-convulsive and other physiologic leukocytoses.

**Year**	**Findings**	**References**
1893	Muscular activity increases the leukocyte	(89)
	count	
1895	Adrenal gland extracts increase blood	(7)
	pressure and cause tachycardia	
1896	GCSs induce transient leukocytoses	(2)
1904	Exogenous adrenaline induces a transient	(12)
	leukocytosis	
1915	Asphyxia, rage and fear release adrenaline,	(9)
	cause tachycardia and redirect peripheral	
	blood to the heart, lungs, and brain	
1942	Rage and fear increase the peripheral	(13)
	leukocyte count in humans	
1952	Adrenaline releases leukocytes and platelets	(25)
	marginated in pulmonary blood vessels	
1955	Increased alveolar pressure favors	(19, 20)
	pulmonary margination	
1959	ECT-induced convulsions increase plasma	(90)
	catecholamines in humans	
1980	PoCL is frequent in CSE and GCSs, but	(52, 54)
	rare in non-motor seizures and PNES	
1981	Most SUDEP autopsies exhibit NPE	(91)
1982	Anaerobic exercise leads to higher	(92)
	adrenaline elevations than aerobic exercise	
1988	Maximal exercise increases leukocyte	(35)
(review)	counts to a greater extent that submaximal	
	exercise	
1990	At any given time 55 - 60% of leukocytes	(21)
	crossing the lungs are marginated inside	
	capillary beds	
1992	GCSs increase plasma catecholamines	(50, 51)
	in humans	
1995	Adrenaline expels leukocytes from the	(26, 29)
	lungs by increasing cardiac output and thus	
	pulmonary blood flow	
2008	Frank PoCL is rare in the EMU but	(65, 68)
	frequent in the ED	
2009	PoCL is associated with death in CSE	(74)
	patients	
2016	Systemic catecholamines also favor	(30)
	demargination by “softening” the PMN	
	cytoskeleton	
2019	The degree of PoCL correlates with	(68)
	aberrant peri-ictal respiration	
2019	PoCL often coexists with NPE	(85)

GCS, generalized convulsive seizure; ECT, electroconvulsive therapy; PoCL, post-convulsive leukocytosis; CSE, convulsive status epilepticus; PNES, psychogenic non-epileptic seizures; SUDEP, sudden death in epilepsy; NPE, neurogenic pulmonary edema; EMU, epilepsy monitoring unit; ED, emergency department; PMN, polymorphonucleocyte.

## Pulmonary margination and physiological leukocytosis

**Figure 1:**
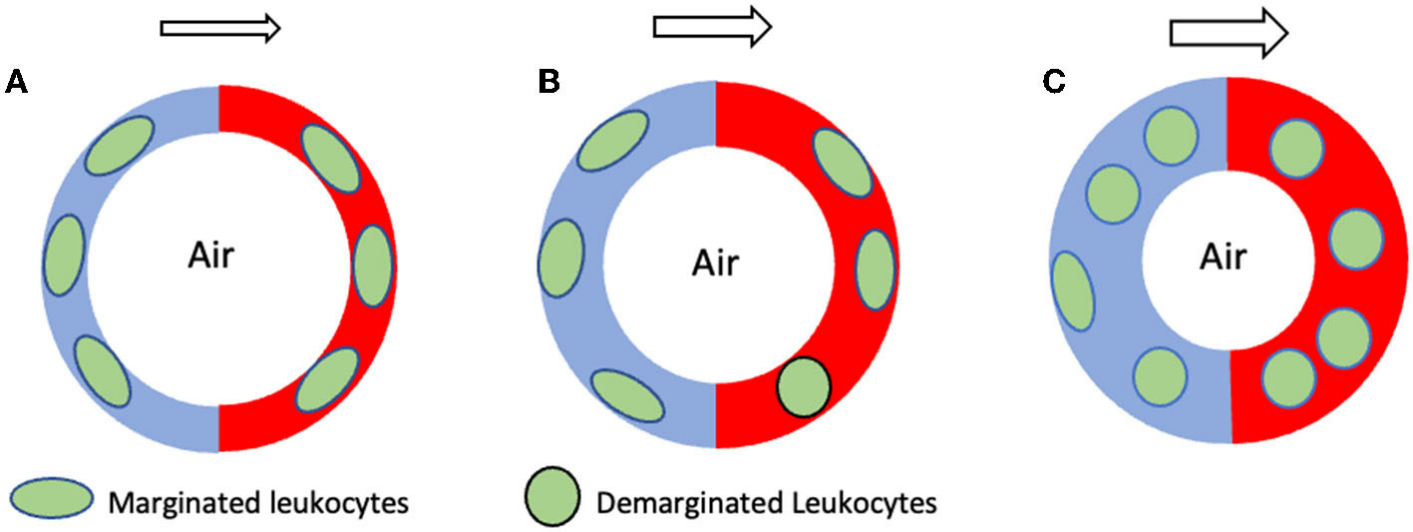
Schematic hypothetical representation of pulmonary demargination during two different seizures. The circular red and blue structures shown represent the collective of all pulmonary alveolar capillaries. The blue and red halves represent the arterial and venous sides, respectively, while the white center represents alveoli. **(A)** Normal, non-seizure state characterized by an equilibrium between margination and demargination and by a normal marginated leukocyte pool. **(B)** During GTCSs exhibiting mostly preserved ventilation only minor increases in blood flow through pulmonary alveolar capillaries occur, resulting in minimal leukocyte demargination and minor increases in the peripheral leucocyte count. **(C)** By contrast, GTCSs exhibiting severe respiratory compromise or apnea drive major increases in blood flow through the pulmonary capillaries, causing major leukocyte demargination and major increases in the peripheral leukocyte count. The arrows above each figure represent the direction of pulmonary blood flow, from arterial to venous. The larger size of the arrows in **(B,C)** represents increased blood flow through the alveolar capillaries during a seizure. GTCS, generalized tonic clonic seizure.

In a parallel line of research, scientists pondered the physiological significance of a large leukocyte pool found inside the pulmonary vasculature which exceeded that of all other organs (16). The origin of this puzzling leukocyte pool turned out to be “margination,” a process characterized by leukocyte crawling onto the vascular endothelial surface before slowing to a halt and remaining out of circulation for up to several minutes at a time (16). While a small fraction of the leukocytes crossing the lungs at any given time marginates inside arterioles and venules, elegant intravital microscopy studies have shown that most pulmonary leukocytes marginate inside alveolar capillaries whose small luminal diameters demand their cytoplasmic transformation from spheres into ellipsoids in order to squeeze through [reviewed by (16–18)]. This sluggish leukocyte transit is further influenced by respiratory mechanics, as, aside from altering the alveolar volume, lung inflations and deflations also alter adjacent capillary diameters. For instance, inhalation attempts against a closed airway (i.e., the Mueller maneuver) decrease intra-alveolar volume, increase alveolar capillary diameter, and promote demargination (i.e., the return of marginated leukocytes into circulation). In turn, these conditions lead to small but detectable elevations in the peripheral arterial leukocyte count. By contrast, exhalations against a closed airway (i.e., the Valsalva maneuver) produce the opposite effects (19, 20). Subtle but similar peripheral leukocyte count elevations and drops can also be observed in the arterial circulation following prolonged exhalations and inhalations, respectively, suggesting the existence of what one scientist in the field called an “ebb and flow of leukocytes” through the cardiopulmonary circulation which is mirrored in the peripheral blood (19). Thus, the so called pulmonary marginated pool (16) arises from a size mismatch between leukocytes and alveolar capillary lumina which delays leukocyte traffic through the lungs (17) (Figure 1). It has been estimated that at any given time 55−60% of all leukocytes crossing the lungs are marginated inside capillary beds (21). Conversely, the more malleable erythrocytes travel through the lungs approximately 60–100 times faster than leukocytes (22), despite also having to alter their shapes while passing through the alveolar capillaries, in single file, exchanging O_2_ and CO_2_ [reviewed by Hogg and Doerschuk (23)].

The polymorphonucleocyte (PMN; also known as granulocyte due to its prominent cytoplasmic granules), the largest and most abundant leukocyte type in the peripheral circulation, which includes neutrophils, mast cells, eosinophils and basophils, is particularly affected by this anatomical peculiarity, and accordingly, its marginated pulmonary pool exceeds its peripheral circulating pool (16). Additional marginated leukocyte pools can be seen inside the spleen, liver, and bone marrow, but their roles in catecholamine- and exercise-induced leukocytoses are less clear. For instance, while some studies suggest that exercise leukocytosis requires the spleen, others show it is unchanged by splenectomy [for review of this literature see (15)]. Marginated leukocyte pools inside the lymphatic system, liver, and bone marrow do not appear to play a significant role in catecholamine-induced leukocytoses (15, 24), although delayed leukocyte elevations associated with long bouts of strenuous exercise stem, at least partially, from cortisol's effects on the bone marrow (see below).

Leukocyte demargination is principally regulated by the speed of blood flow, as shear force alone is sufficient to sweep leukocytes out of the alveolar capillaries (16, 21, 25–29). Consequently, systemic catecholamines, whether endogenous or exogenous, promote demargination primarily by increasing heart rate, which expands blood volume and accelerates blood flow through the pulmonary vasculature (26, 29). In addition, systemic catecholamines further facilitate leukocyte demargination by “softening” the leukocyte cytoskeleton (30) and by hindering leukocyte adhesion to the alveolar capillary endothelium (31). A physiological effect of systemic catecholamines on the blood leukocyte count is illustrated by studies in healthy humans [see Garrey and Bryan (32) for a review of this literature] which demonstrated that mere physical activity elevates the leukocyte count to a degree that depends on exertion (32) and heart rate (33). Maximal intensity exercise increases both systemic catecholamines [reviewed by Zouhal et al. (34)] and blood leukocyte counts more efficiently than submaximal exercise [reviewed by McCarthy and Dale (35)]. Additional evidence of a direct relationship between systemic catecholamines and the peripheral leukocyte count can be found in myriad reports of conditions in which either increased heart rate or frank tachycardia occur, including transient hypoglycemia (36), acute trauma (27), symptomatic pheochromocytoma (37), amphetamine use (38), atrial fibrillation (39), acute burns (40), obstructive sleep apnea (41), acute stroke (42), myocardial infarction (43), thyroid storm (44), and others (45, 46). It should be emphasized that, while physiological leukocytoses have *hypothetical* immunological consequences (15), investigations that automatically ascribe proinflammatory roles to sudden, unexpected, and transient elevations in the peripheral leukocyte count without contemplating the effects of catecholamines and heart rate should be interpreted with caution.

## Post-convulsive leukocyte elevations

Even though PoCLEs are considered physiologic (47, 48), their underlying mechanisms remain mostly unexplored. Animal and human studies have documented an intense activation of the sympathetic nervous system [e.g., (49)] and a release of systemic catecholamines immediately after GCS (50, 51), but these studies have not investigated their direct effects on the peripheral leukocyte count. Instead, most of what is known about PoCLEs comes from small observational and retrospective clinical investigations. For instance, an epilepsy monitoring unit (EMU) study of 340 epileptic seizures in 89 patients showed PoCLEs in 36% of GCS, 7% of complex partial seizures and 0% of non-convulsive or psychogenic non-epileptic seizures (PNES; formerly known as pseudoseizures) (52). PoCLEs exceeding the upper range of the normal leukocyte count have been frequently observed in convulsive status epilepticus (CSE) patients [e.g., 41.6% (53), 62.5% (54)], suggesting that convulsive intensity and duration influences the degree of these elevations. This notion, which is as old as Joseph Capps' first description of PoCLEs (2), has been both embraced and refuted throughout the last century. Just 20 years after Capps claim that the degree of PoCLEs correlates with the “length and severity of attacks”, a Philadelphia researcher wrote in The Lancet: “…the degree of increase in the leukocytes [does not] bear any relationship to the severity of the convulsive paroxysm” (55). A more recent emergency department (ED) study of 203 pediatric febrile seizures also failed to find an association between convulsive duration and leukocyte counts (56). However, that study omitted the longest and most severe GCSs from statistical analyses, as the authors excluded GCSs that met their definition of CSE (i.e., continuous seizures or repeated convulsions without recovery of consciousness lasting 30 min or longer) (56). By contrast, an EMU study in which the time elapsed between convulsions and blood collections was controlled for, found a significant correlation between the duration of convulsions and the degree of PoCLEs (52). Consequently, whether convulsive intensity and duration directly affect the post-convulsive leukocyte count remains an open question.

## Role of cortisol

Administration of exogenous catecholamines induces an immediate lymphocyte peak (within 30 min) followed by a delayed PMN peak (within 2–4 h) (15). By contrast the administration of exogenous steroids induces a gradual increase of PMNs over several hours (57) by accelerating their release from the bone marrow, increasing their circulating half-life, and reducing their egress from the intravascular compartment [reviewed by Parillo and Fauci (58)]. This slow steroid response is consistent with its intracellular mechanism of action, which involves a multi-step process that in some cases includes gene transcription (35). Cortisol's effect on PoCLEs has not been directly investigated, but studies show that following GCSs cortisol is released slowly, marginally and inconsistently (59, 60). At least one study suggests that cortisol's effects on the post-convulsive leukocyte count follow those of catecholamines, as its release peaked 30 min after GCSs and returned to baseline within 120 min (61). In addition, early animal work demonstrated a leukocyte peak with a left shift (i.e., the presence of bone marrow-derived, immature PMNs) 4 h after convulsions in 6-OHDA and reserpine treated animals (62), suggesting it was instigated by a non-catecholaminergic stimulus such as cortisol on the bone marrow. Thus, if or when cortisol is released after GCSs, it is likely to augment the initial effects of catecholamines on the peripheral leukocyte count.

## Kinetics and cellular composition

The essence of what is known about PoCLE kinetics was written by Joseph Capps in his original publication at the end of the 19th century (2), namely that GCSs induce leukocyte elevations which start during or immediately after convulsions and resolve within approximately 24 h. Burrows, in 1899, observed that some PoCLEs involved a gradual increase in PMNs for several hours after convulsions. For instance, 40 min after a GCS one of his patients' leukocyte counts was 13,000 cells/mm^3^ out of which 70% were PMNs, but 4 h later his leukocyte count was 16,500 cells/mm^3^ out of which 91% were PMNs (3). Decades later, studies of electroconvulsive therapy demonstrated an early increase in lymphocytes within 3 min of convulsions which returned to baseline within 15 min. During this lymphocytic increase, PMNs exhibited “violent fluctuations,” increasing in some patients and decreasing in others (63). In rabbits, cardizol-induced GCSs showed an immediate and fleeting lymphocytic peak followed by a steady rise in PMNs which lasted several hours (64). A more recent EMU investigation in which the average time between convulsions and blood collections was 10 ± 6.0 min, demonstrated significant leukocyte elevations within the normal range. These elevations consisted of relative increases in both lymphocytes [natural killer (NK)-like T cells] and PMNs (neutrophils), which returned to baseline within 24 h (65). Of fifty infection-free CSE patients who demonstrated abnormally increased leukocyte counts at the time of hospital admission (range 12,700–28,800 cells/mm^3^) 34 and 22% showed significant increases in PMNs and lymphocytes, respectively, while the rest showed normal differential counts (54). In the aggregate, PoCLEs appear to involve rapid and short-lived lymphocyte increases followed by slow and steady PMN increases that return to baseline within ~24 h.

## Role in clinical practice

Physicians and other clinicians typically encounter PoCLEs in patients who present to EDs with GCSs and abnormally elevated leukocyte counts. As these patients' medical histories and workups lack common leukocytosis triggers such as therapeutic corticosteroids, infection, or lymphoproliferative disease, the physiological nature of these leukocytoses only becomes apparent in retrospect, after subsequent blood draws reveal their spontaneous normalization. Moreover, extensive early investigations negated the existence of a correlation between baseline leukocyte counts and epilepsy *per se* (66, 67) and therefore it is not surprising that post-convulsive leukocytoses are often perceived as mere seizure epiphenomena of little clinical value whose apparently random appearance forces treating clinicians to embark on fruitless searches for infectious sources (53, 56). In addition, PoCLEs are often misunderstood as occurring strictly above the normal leukocyte range, even though most GCSs, especially those which do not require urgent transport to the ED, probably induce PoCLEs within the normal leukocyte range. For instance, in patients admitted electively to an EMU, average PoCLEs occurred entirely within the normal leukocyte range (from 5,900 to 8,330 cells/mm^3^; normal range 4,000–11,000 cells/mm^3^) (65). Recently we estimated that 89.5% of 105 patients hospitalized with GCSs experienced PoCLEs either within or above the normal leukocyte range (68). Therefore, paraphrasing from an early publication (55), it is likely that GCSs in otherwise healthy patients invariably cause temporary elevations of the leukocyte count. Yet, despite their frequent presence in emergency settings, the clinical significance of PoCLEs remains obscure. Animal studies have shown a breakdown of the blood brain barrier following status epilepticus [reviewed by Swissa et al. (69)] and a lymphocytic infiltration of neocortex and hippocampus following maximal electrically induced seizures (70). The relationship between these findings and PoCLEs has not been explored. It is possible, however, that some of the leukocytes released during PoCL could infiltrate the brain, but the consequences of such infiltration are unclear. Some investigators have proposed that leukocytosis and other concomitant effects of CSE such as fever, acidosis, and hypoxemia (54, 71) could help differentiate generalized CSE from intractable PNESs in emergency settings (72), a notion supported by the negligible effect of PNESs' on the peripheral leukocyte count (52). A small retrospective investigation suggested that bloodwork collected within 9 h of GCSs can be used to differentiate epileptic seizures from PNESs through the following equation: [(1.5 × anion gap) + (leukocyte count)]. While this method has not been validated prospectively, the authors indicated that a result ≥24.8 or ≤ 15.5 confers a ≥90 or ≤ 10% probability, respectively, that the seizure in question is epileptic (73).

In a different line of investigation, Tiamkao and Sawanyawisuth studied predictors of death in 32 cases of generalized CSE treated with sodium valproate and concluded that, when found at presentation, post-convulsive leukocytosis was associated with death (74). Recently, one of these authors (JLV) and colleagues, found a significant correlation between the degree of PoCLEs and the presence of periconvulsive signs of respiratory distress (68). As that study also revealed a statistically significant correlation between post-convulsive leukocyte counts and ED triage heart rates, it was hypothesized that aside from inducing PoCLEs catecholamines might play a role in producing, or in exacerbating, periconvulsive respiratory symptoms. The latter could result from various degrees of transient neurogenic pulmonary edema (NPE) (75–77) a frequent and sometimes recurrent (78–81) periconvulsive finding thought to be at least partially driven by catecholamine-mediated increases in pulmonary blood flow and vascular tone [reviewed by (82–84)]. This proposed mechanism is consistent with a recent report that NPE and leukocytosis often coexist (85). Considering the close relationship between pulmonary marginated leukocytes and cardiopulmonary circulation dynamics, these data suggest that oxygen deficits generated by periconvulsive respiratory aberrations such as central apnea, inefficient respiratory mechanics, airway obstruction, or laryngospasm [reviewed by Stewart et al. (86)], which at times result in death (i.e., SUDEP) (1), could at least partially contribute to different degrees of NPE heralded by the post-convulsive leukocyte count. Viewed through this lens, the unpredictable relationship between PoCLEs, convulsive duration and convulsive intensity, so frequently highlighted during the last century, could be explained by shifting cardiopulmonary blood flow dynamics occurring in the context of oxygen-conserving reflexes, such as the mammalian diving response, whose sympathetic arm shunts a significant portion of the total blood volume toward the cardiopulmonary vasculature [reviewed in (87)]. Thus, understanding the relationship between periconvulsive respiratory anomalies, periconvulsive cardiopulmonary circulation dynamics, and PoCLEs not only has the potential to reveal important clues about SUDEP pathophysiology, but also about SUDEP risk and SUDEP diagnosis. For instance, an in-depth understanding of periconvulsive leukocyte kinetics could provide the post-convulsive leukocyte count a new role as a marker of underlying respiratory pathology. Parallel efforts to elucidate whether PoCLEs, or drops, depending on whether convulsive semiology favor margination or demargination, demonstrate patient-specific patterns (e.g., amount of time elapsed between convulsions and leukocyte changes, degree of leukocyte change, change in the differential leukocyte count, etc.) could be used to stratify SUDEP risk. In a different investigative vein, timed histological analyses of marginated leukocyte pools from SUDEP victims could bring us closer to a tissue based postmortem SUDEP diagnosis. Therefore, in certain patients, PoCL has the potential to serve as a biomarker for SUDEP and SUDEP risk. Unfortunately, little is known about the post-convulsive leukocyte counts of SUDEP victims, or even of near-SUDEP patients (i.e., SUDEP victims who are resuscitated and survive for 1 h or longer) as their leukocyte counts are seldom reported. In a rare exception, Christy et al., described the near-SUDEP experience of an 11-year-old patient with Lesch Nyhan syndrome who developed respiratory failure and required emergent intubation following a first-of-life GCS. His post-convulsive leukocyte count was 32,000 cells/mm^3^ and his workup failed to demonstrate an infection (88).

## Conclusion

One hundred and twenty-six years after their discovery, PoCLEs remain a physiological enigma. While the available literature confirms Joseph Capps' original conclusions that they are transient, non-infectious, redistributive increases in the peripheral leukocyte count provoked suddenly by epileptic convulsions, it also shows a pervasive tendency to assume that PoCLEs are driven by the same mechanisms underlying catecholamine and exercise induced leukocytoses. Yet, unlike other physiological leukocytoses, PoCLEs are triggered during unpredictable combinations of involuntary muscle contractions and aberrant respiratory mechanics which can result in impaired ventilation, impaired gas exchange, or both. Given the intimate relationship between the pulmonary marginated leukocyte pool and the cardiopulmonary circulation, elucidating the mechanisms by which individual seizures instigate PoCLEs could prove essential in our quest to understand, prevent, and diagnose SUDEP.

## Author contributions

All authors contributed to the research and writing of this review.

## Conflict of interest

Author JLV is the founder and owner of TeleNeurologia SAS.

The remaining authors declare that the research was conducted in the absence of any commercial or financial relationships that could be construed as a potential conflict of interest.

## Publisher's note

All claims expressed in this article are solely those of the authors and do not necessarily represent those of their affiliated organizations, or those of the publisher, the editors and the reviewers. Any product that may be evaluated in this article, or claim that may be made by its manufacturer, is not guaranteed or endorsed by the publisher.
